# Machine learning-based forecasting of rainfall and water demand for urban water planning: the case of Ekurhuleni, South Africa

**DOI:** 10.1038/s41598-026-51831-1

**Published:** 2026-05-06

**Authors:** Murphy Bonkogia Lomboli, Opeyeolu Timothy Laseinde, Clinton Ohis Aigbavboa

**Affiliations:** 1 Department of Mechanical Engineering Science, University of Johannesburg, Johannesburg, South Africa; 2 Department of Mechanical and Industrial Engineering Technology, University of Johannesburg, Johannesburg, South Africa; 3 Department of Construction Management and Quality Surveying, University of Johannesburg, Johannesburg, South Africa

**Keywords:** Urban water demand forecasting, Rainfall forecasting, Machine learning, Gaussian process regression, Time-series forecasting, Climate sciences, Engineering, Environmental sciences, Hydrology

## Abstract

This study develops and evaluates a data-leak-safe, monthly forecasting framework for the City of Ekurhuleni, South Africa, covering rainfall and municipal water demand. Here “leak-safe” means that all predictors are built from information that would have been available at the forecast month, with feature engineering, scaling and model validation performed strictly on the training window only. The results are situated within demographic change, addressing the gap in decision-grade monthly forecasts that jointly consider rainfall and municipal demand for planning. Monthly datasets (2011–2025) were cleaned and engineered using past-only features (fixed lags; trailing 3/6/12-month statistics; harmonic month terms; simple trend). Models were trained using MATLAB with 5-fold cross-validation (PCA capped at 95% variance when applied) and benchmarked against persistence, seasonal-naïve, and monthly climatology on a sealed test window. For rainfall, a bagged-trees ensemble achieved strong generalization (test RMSE ≈ 9.13 mm; R^2^ ≈ 0.96), capturing wet-season peaks (Dec–Feb) and dry-season minima (Jun–Aug). For demand, a Matérn-5/2 Gaussian Process delivered positive out-of-sample skill (test RMSE ≈ 17.05 ML/day; R^2^ ≈ 0.76; MAPE ≈ 1.39%), tracking month-to-month movements with mild amplitude damping. A 36-month recursive rollout indicates stable consumption within a narrow band (approximately 995–1025 ML/day) and a seasonal rainfall envelope consistent with historical patterns. Census-based trends, growth in formal residential areas, and increased in-dwelling/yard tap access support a rising, more metered base load with localized variability. The synthesis suggests prioritizing reliability, active leakage control, targeted equity upgrades, and routine re-forecasting over large capacity expansion, while using rainfall-conditioned scenarios and uncertainty bands for procurement and risk planning. The contribution is a reproducible, decision-grade pipeline that pairs rigorous baselines with actionable 36-month forecasts for urban water resources management.

## Introduction

### Background and motivation

Globally, water resources management has become a crucial concern particularly in the wake of increasing urbanization, compounded uncertainties in terms of climate change and the rapidly growing populations. Urban areas, particularly in the Global South, are facing complex challenges in maintaining a balance between economic development and sustainable access to water. Both factors which are highlighted in the United Nations (UN) Sustainable Development Goals (SDGs) 6 and 11^1^. Global projections estimate that by the year 2050, nearly 70% of the world’s population will reside in urban areas (a growth by 2.5 billion urban dwellers between the year 2018 and 2050), with nearly 90% of the increase being concentrated in Asia and Africa^2,3^.

South Africa represents a microcosm of this globally recognised challenge. As a semi-arid country with limited access to freshwater and further challenged by the uneven spatial distribution of water resources, it ranks among the driest countries in the world^4^. Its history of spatial inequality, combined with urban sprawl and informal settlements, particularly in the Ekurhuleni metropolitan area among others, adds additional strain on water supply and planning systems^5^. The Ekurhuleni municipality in the Gauteng Province has developed into a strategic industrial and logistics hub, that is home to roughly three to four million people and rapid growth in both formal and informal settlements has increased water consumption and pressure on municipal services^6,7^. To add to this, the rapid settlement expansion often outpaces infrastructure upgrades, this can create disparities in water access and delivery reliability.

Climate change has additionally aggravated these challenges. South Africa, including the Gauteng province has experienced increased variability in rainfall patterns, which leads to periods of drought interspersed with flooding events. Such inconsistencies can undermine long-term planning thereby threatening water security^8,9^. As highlighted in recent studies, future rainfall projections and the availability of water in South Africa remains challenging, calling for predictive and adaptive resource management strategies^10^.

In this context, the use of machine learning and predictive analytics as shown in numerous studies, have emerged as valuable tools for forecasting water demand, rainfall variability and settlement trends. While traditional statistical methods/models often fail to relay the nonlinear relationships of urban systems, advanced models like dynamic Artificial Neural Networks (ANN) and Ensemble Learning Techniques (ELT) can offer improved accuracy as well as adaptability^11^. These technologies can enable policymakers and planners to simulate and test numerous future scenarios for improved resource allocation.

Moreover, the integration of spatial land use dynamics such as changes in Enumeration Area (EA) type (e.g., formal, informal and traditional residences), into predictive models can enhance the granularity and relevance of urban planning decisions. As highlighted in the study by^12^, the harmonizing of water and spatial planning is key to achieving more equitable and resilient cities in the 21 st century. Recent studies have also linked demand characterization directly to infrastructure sizing and flood-mitigation design in African cities, for example through retention-basin analysis in Ouagadougou and specific-consumption-based design of drinking-water systems in Burkina Faso^13,14^. This aligns with broader findings that accurate characterization of specific consumption and peak behaviour is central to economically robust sizing of urban water and flood-mitigation infrastructure in resource-constrained settings.

The motivation behind this study arises from the need to develop a localised, data-driven forecasting model that can independently predict trends in rainfall and future water demand in Ekurhuleni. This aligns well with SDG 6 on clean water and sanitation, particularly expanding access to safe and affordable drinking water and improving water-use efficiency as well as SDG 11 on sustainable cities and communities, which emphasises more inclusive, participatory urban planning and human settlement management.

This study makes three practical contributions:

- A data-leak-safe feature design for monthly rainfall and demand. Predictors were built from past information only which included fixed lags, trailing means and standard deviations that stopped at (t-1), harmonic seasonality, a month index and regime flags plus a past-only monthly climatology as well as anomaly target option for robustness.
- Rigorous benchmarking and reporting. The models were evaluated against persistence (t-1), seasonal-naïve (t-12) and established monthly climatology baselines. We report standard error metrics on held-out periods, including Mean Squared Error (MSE), Root Mean Squared Error (RMSE), Mean Absolute Error (MAE), Mean Absolute Percentage Error (MAPE), Normalised RMSE (NRMSE) and the coefficient of determination (R^2^) so that performance is interpretable for operations.
- Actionable 36-month forecasts, produced on recursive monthly forecasts for rainfall and municipal consumption with a reproducible MATLAB workflow which is suitable for planning, scenario analysis and communication with relevant stakeholders.

### Problem statement

Urban municipalities in South Africa, are increasingly under pressure to meet the growing demand for essential services like water, housing and sanitation in a context that is often marked by erratic climate conditions and service delivery backlogs. While spatial expansion and population growth are well-documented, there is a lack of integrated tools that could accurately predict how these dynamics interact to influence water demand and planning of infrastructure.

Ekurhuleni, like many fast-urbanizing municipalities within the republic, faces challenges of managing unpredictability in rainfall patterns intensified by climate change, whilst ensuring a reliable water supply amidst evolving settlement patterns. The increasing demand on limited water resources, driven by factors such as population growth, highlights the urgent need for predictive tools that can support evidence-based urban water planning and infrastructure management.

### Research objectives

The overarching aim of this research is to develop a predictive framework that separately forecasts monthly rainfall and municipal water demand for the Ekurhuleni Municipality. The study models rainfall and demand independently so that each model can be tuned to the characteristics and constraints of its own historical series, while still allowing their outputs to be interpreted together for planning.

The core objectives of the study are:

- Understanding potential future changes in water availability caused by climate variability. This was done by forecasting rainfall patterns for the Ekurhuleni region using historical meteorological data and machine learning regression techniques.
- Future demand, projecting future water demand/consumption trends using historical data from utility partners.
- Validating the accuracy and performance of each predictive model using established metrics such as Root Mean Square Error (RMSE), Mean Square Error (MSE), and R^2^.
- To synthesize the independently predicted outputs and assess their interdependencies. In this first implementation, rainfall and demand are forecast as separate univariate series and then interpreted jointly in the planning discussion, rather than forcing rainfall directly into the demand model; we treat the use of rainfall anomalies as explicit exogenous demand predictors as a natural extension for future work.

### Forecasting in water resources management

Forecasting in water resources management, especially with regard to urban areas, is increasingly becoming vital due to rapid urbanization, resource constraints and climate variability as discussed. Globally and locally, decision-makers require reliable predictive models in order to anticipate rainfall patterns, settlement expansion and future water demands^15,16^.

In recent years, there has been a shift from conventional statistical methods, such as time-series models, to Artificial Intelligence (AI)-driven techniques, particularly Machine Learning (ML) and hybrid models, to improve the accuracy and scalability of water demand forecasting^17^. These approaches are preferred for their capacity to model nonlinear relationships whilst integrating multiple datasets and generate real-time predictions^18^.

Within South Africa several published studies have highlighted the significance of predictive modelling in enhancing water security, particularly for urban and peri-urban regions. For example, using machine learning in potential groundwater mapping and rainfall runoff has become common and shown to help improve reliability in resource planning^19^.

Moreover, AI-based models/tools have also been used for spatiotemporal modelling of water quality^20^, AI-based approaches enable localized predictions that are critical for public health and infrastructure maintenance. Additionally, integrated frameworks that incorporate land use, population growth and rainfall variations into a more unified forecasting system are gaining traction globally and therefore offer promising results for urban centres like Ekurhuleni.

Beyond South Africa, recent work in northern Australia has shown how machine-learning and hybrid models can support decision-grade rainfall forecasting at seasonal and wet-period scales. Farooq and co-authors use Artificial Neural Networks and Random Forest models driven by lagged large-scale climate indices such as the Indian Ocean Dipole, Southern Oscillation Index, Interdecadal Pacific Oscillation and Madden–Julian Oscillation to forecast wet-season rainfall at multiple stations in the Northern Territory and report clear gains over multiple-linear-regression baselines^21^. They also develop wavelet-based hybrid schemes for seasonal rainfall and multi-algorithm frameworks that treat climate indices as predictors^22,23^, and explicitly compare Random Forest with SARIMA for wet-period prediction, where the non-linear models generally outperform the classical time-series formulation once teleconnection signals are included. Together these studies reinforce that combining climate indices, time-frequency decompositions and tree-based learners can yield robust rainfall forecasts in semi-arid environments, and they provide a useful global reference point for the simpler, past-only rainfall model adopted here for Ekurhuleni.

Moreover, Neural Network-based tools such as Long Short-Term Memory (LSTM), Dynamic Artificial Neural Networks (DANN), and Genetic Programming-enhanced Extended Kalman Filters have shown much higher performance over traditional statistical models when dealing with large and multivariate datasets involving consumption and settlement changes^24^.

Below is a tabulated summary of some of the most notable studies that illustrate the progression of forecasting techniques in urban water management.

**Table 1:** Summary of some of the studies in urban water forecasting.

Study	Method	Target & timescale	Location	Best Metric	Key Insight
Forecasting daily urban water demand: a case study of Melbourne^25^	Decomposition time series	Daily water demand	Melbourne, Australia	R^2^ ≈ 0.896; Standard Error ≈ 8%	Accurate short-term demand prediction: model adapts to variable input conditions
Hybrid regression model for near real-time urban water forecasting^24^	Hybrid Machine Learning	Real-time near forecasting	Franca, Brazil	RMSE = 1.318, MAE = 3.45%, R^2^=0.974	Real-time adaptation to system changes, optimal retraining cycle balances accuracy vs. computation
Artificial Neural Network (ANN) - Based Water Quality Index (WQI) for Assessing Spatiotemporal Trends in Surface Water Quality - A Case Study of South African River Basins^20^	ANN	Water quality index	KwaZulu-Natal Province, South Africa	*R* = 0.985, R^2^=0.970, NSE = 0.974	Effective for detecting pollution and health risk trends
Probabilistic analysis of water quality deterioration and health risks assessment incorporating machine learning techniques in Mhlathuze catchment, South Africa^26^	Logistic Regression, Random Forest, XGBoost, Decision Tree, K-Nearest Neighbors	Water quality assessment, pollution source identification, and health risk evaluation	Mhlathuze catchment, South Africa	Accuracy, Precision, Recall, F1 score of algorithms: LR, RF, XG, DT and KNN were reported	Combines spatial/temporal data for localized risk assessment
Forecasting monthly urban water demand using Extended Kalman Filter and Genetic Programming^27^	Genetic Programming (GP) with Extended Kalman Filter (EKF)	Monthly water use	Tehran, Iran	NMSE = 0.085, R^2^=0.94 (GP + EKF)	Useful in nonlinear time series and seasonal adjustment contexts

Together, these studies show a clear shift toward machine-learning approaches in water resources planning, particularly for short-term, high-frequency forecasting at plant or city-sector level. However, most of the high-performing models are either daily or hourly, focus on single systems, and rarely report multi-year, monthly out-of-sample performance against simple baselines. This study extends that work by focusing on monthly, city-scale rainfall and demand forecasting for a bulk-supplied metro, with transparent data-leak-safe validation and explicit comparison to persistence, seasonal-naïve and climatology benchmarks.

### Gaps in existing work

Firstly, there are very few peer-reviewed demand-forecasting studies in South African metros that are utility-scale evidenced and none identified in this review that is specific to Ekurhuleni with its bulk-supply. The broader literature also points out to two cross-cutting holes: limited use of probabilistic forecasts and inconsistent, transparent post-evaluation of deployed models. Both of these issues matter for planning buffers and investment risk in a city like Ekurhuleni where supply is purchased rather than self-produced^28,29^.

Secondly, many of the high-performing studies are short-term and plant-specific (hourly/daily), often showing strong gains from hybrid or neural methods but they don’t transfer cleanly to medium-term monthly horizons that could be used for budgeting and demand management.

For example, hybrid SOM+Regression-Tree models beat SARIMA in short-term tests and yet the papers emphasize operational horizons without reporting multi-year, monthly out-of-sample tests against rigorous baselines like persistence and seasonal-naïve. This therefore leaves a gap for medium-term municipal planning where seasonality and structural shifts dominate^30,31^.

Lastly, advanced ANN architectures such as DAN2 tend to achieve very high accuracy across multiple horizons, but they are typically tested on single-system case studies with stable contexts and limited emphasis on uncertainty, reproducible baselines or municipal integration constraints such as bulk-purchase rules, pressure zones and demand-curtailment stages. This creates a gap for city-level monthly forecasting frameworks that generalize across regime changes and report performance against simple operational baselines^11,18^.

## Methodology

### Study area description

**Fig. 1:**
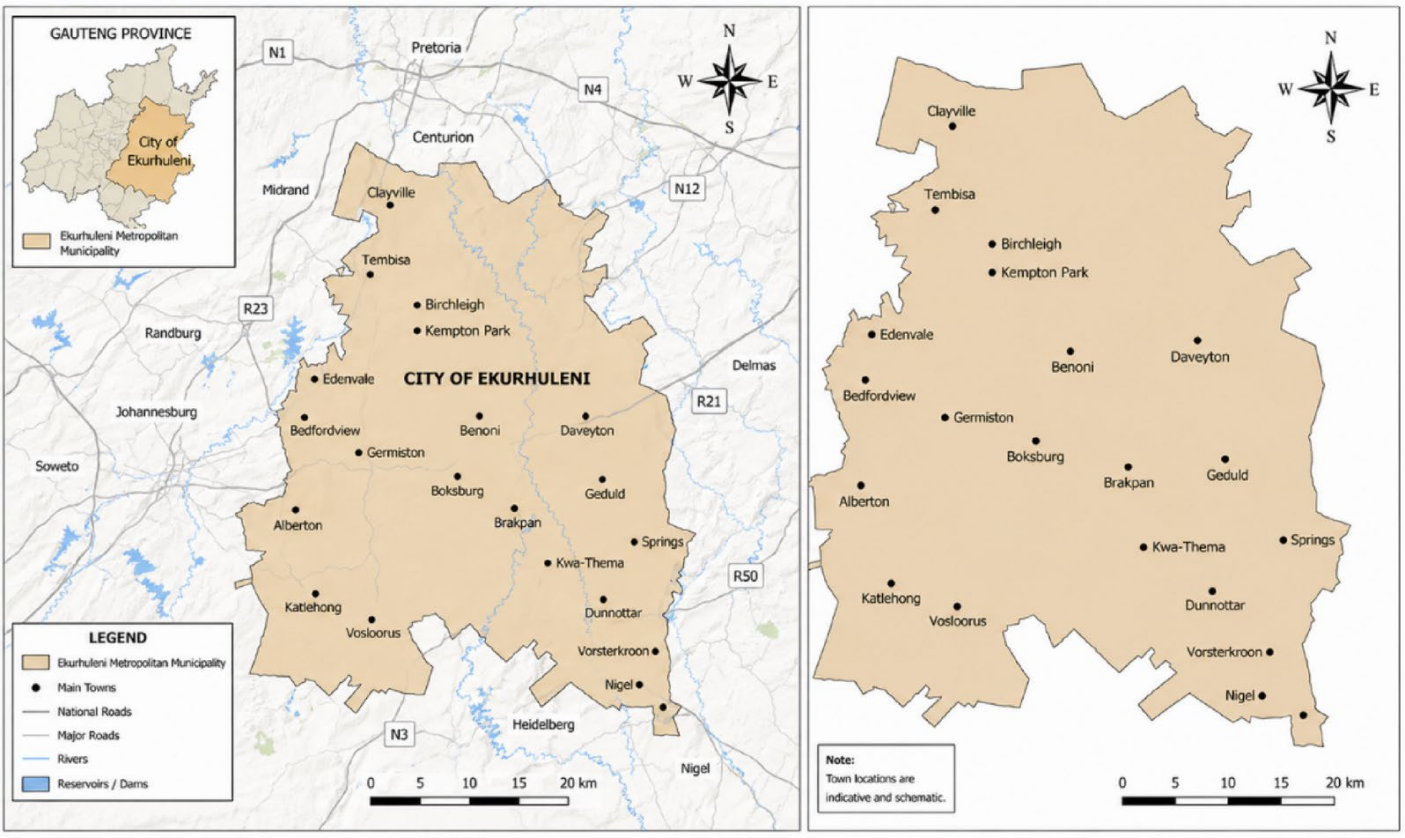
(**a**) Study area map; (**b**) Spatial distribution of major towns in the City of Ekurhuleni, South Africa. Source: Author’s compilation using^34,35^.

The Ekurhuleni Metropolitan Municipality is situated on the eastern flank of the Gauteng City Region as shown in Fig. 1a and covers roughly 1 975 km^2^. It is one of three metros in the Gauteng Province and is densely urbanized and housed about 4.07 million people in 2022 with a population density around 1 609 people/km^2^. The metro accounts for roughly 27% of Gauteng’s population and about 7% of South Africa’s population^32^.

Historically the metro consolidated nine former municipalities namely: Alberton, Edenvale, Kempton Park, Germiston, Boksburg, Benoni, Brakpan, Springs and Nigel, shown in Fig. 1b, whose growth followed the Witwatersrand gold reef. This legacy produced multiple CBDs, extensive industrial belts and large township areas like Tembisa, Kathorus, Kwa-Tsaduza and Daveyton-Etwatwa, connected by rail and freeway corridors that still shape travel and service patterns today^33^.

In Fig. 1b the main towns in the city (e.g. Alberton, Edenvale, Kempton Park, Germiston, Boksburg, Benoni, Brakpan, Springs and Nigel) broadly align with the main service clusters and pressure zones in the metro. The industrial belts around Germiston-Boksburg-Benoni and the dense township areas such as Thembisa host a large share of residential and industrial activity and therefore a large share of municipal water demand. While the present models use spatially aggregated rainfall and consumption at metro level, Fig. 1b highlights the sub-areas where future zone-level forecasts, strategic placement of additional gauges in key supply catchments and targeted infrastructure upgrades could be most critical.

**Fig. 2:**
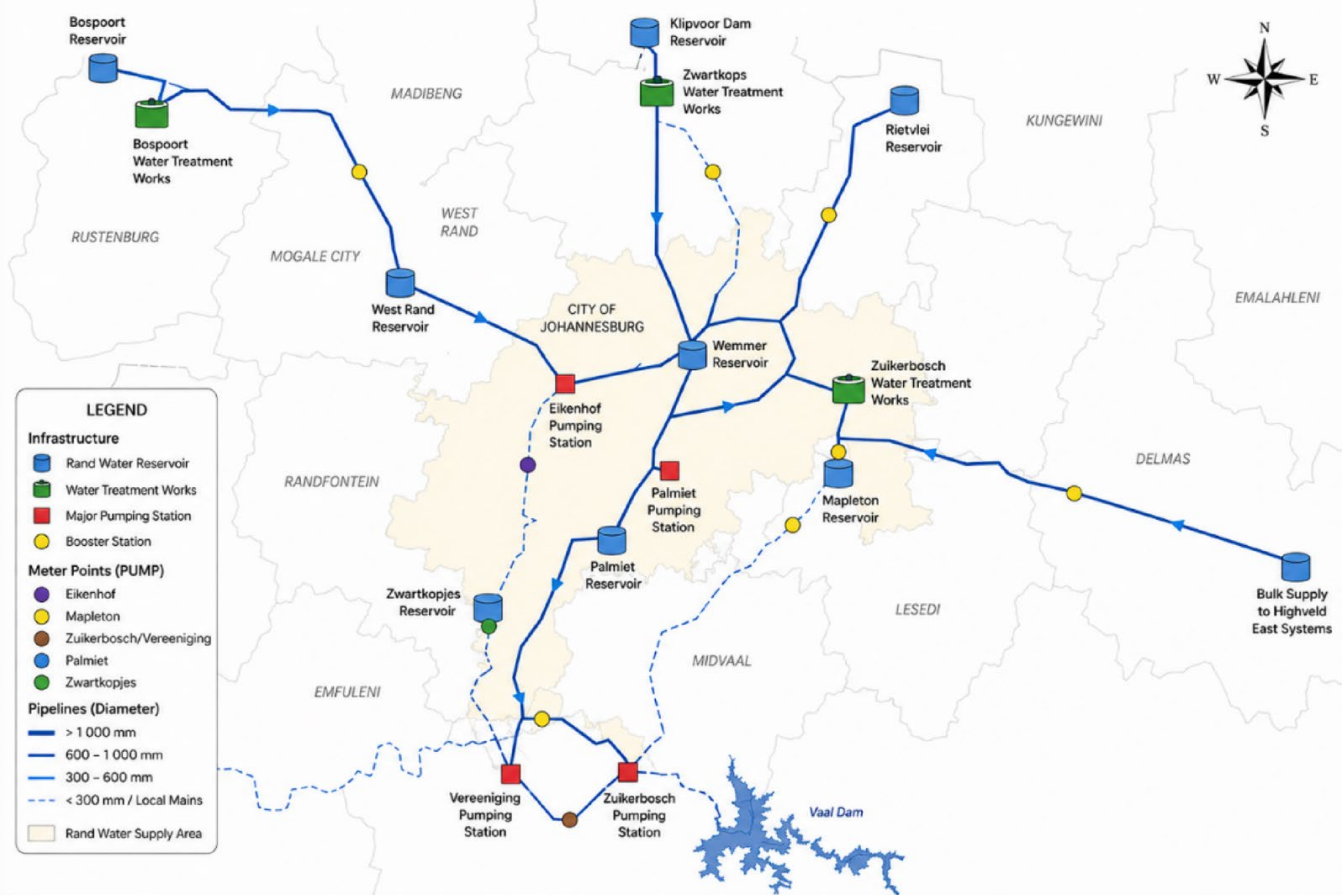
Schematic representation of Rand Water supply system (author’s illustration based on^38^.

The City is both the Water Services Authority and the Water Services Provider under the Water Services Act. As such, it must ensure access to potable water and sanitation within its jurisdiction, ensuring operation and maintenance of the local distribution infrastructure. These duties are carried out subject to resource availability, equitable allocation and demand management^34,35^. Bulk potable water is supplied to Ekurhuleni primarily by Rand Water. Rand Water abstracts raw water mainly from the Vaal River System, treats it at the Vereeniging and Zuikerbosch plants, and distributes it through a regional backbone that includes about 3 500 km of pipelines, four major booster stations (Zwartkopjes, Palmiet, Eikenhof and Mapleton) and multiple reservoirs as shown in Fig. 2. The Mapleton and Palmiet systems serve significant portions of Ekurhuleni^36,37^.

Typically, Rand Water dispatches the bulk of treated water from Zuikerbosch and Vereeniging to the booster network with an approximate average daily delivery on the order of Zwartkopjes 760 ML/day, Palmiet 1 860 ML/day, Eikenhof 1 200 ML/day and Mapleton 730 ML/day. These nodes raise pressure and then provide secondary disinfection and feed municipal reservoirs and zones across the Gauteng Province, including Ekurhuleni^36^.

Recent capacity and reliability projects are targeted at the Ekurhuleni supply corridor. The Mapleton upgrade adds 600 ML/d of pumping capacity, benefiting areas such as Brakpan and Benoni, and Rand Water has deployed mobile emergency generators at Mapleton and is rolling out similar resilience measures at Palmiet, Eikenhof and Zwartkopjes to maintain supply during major power failures. At municipal level, Ekurhuleni reports ongoing service delivery actions to secure household access, including new metered connections and additional reservoirs, alongside compliance monitoring of trade effluent to protect water quality downstream. These initiatives are aimed at maintaining reliable potable supply across formal and informal settlements as the population grows.

### Methods and tools used in related studies

As an overview, Machine Learning is a subsection of Artificial Intelligence. Primarily machine learning is categorized into: Supervised Learning, Unsupervised Learning and Reinforcement Learning. Supervised learning is typically used when the goal is to predict a known target variable (e.g., water quality index, river flow, water demand) set based on input features. Some of the well-known examples of supervised learning: Artificial Neural Networks (ANNs), Support Vector Machines (SVMs) and Support Vector Regression (SVR), Decision Trees and Ensemble Methods, Linear and Non-Linear Regression^39,40^.

Unsupervised learning on the other hand is typically used to find inherent structures or patterns within data without a pre-defined target variable. Some examples of Unsupervised learning: Clustering Algorithms (CA), Principal Component Analysis (PCA). Whereas Reinforcement Learning involves learning optimal actions through trial and error. In water resources management, Supervised and Unsupervised Learning are typically used, more so than reinforcement learning. Explained below are some of the commonly used techniques in water resources management, of these, selected, if not all were used in training, validation, testing and predictions made in this paper.

#### Artificial Neural Networks (ANNs)

Artificial Neural Networks (ANNs) are inspired by the human brain and consist of layers of interconnected neurons that transform inputs through weighted sums and nonlinear activation functions. They are particularly useful for capturing nonlinear relationships between rainfall, temperature, socio-economic drivers and water demand, which traditional linear models may miss. In this study ANNs are used as one of several benchmark learning methods; the detailed forward-pass, activation and backpropagation formulations follow standard ANN training practice.

In urban water studies, ANNs have been repeatedly applied to short-term demand forecasting and have often outperformed classic regression/time-series baselines, including a well-known campus case where the best ANN achieved approximately 2.4% average absolute error^41^. Broader academic reviews have also noted the rise of backpropagation ANNs in water-demand modelling alongside other ML methods^42^.

In neuron aggregation + activation (forward pass):

1 $$\:{net}_{j}=\sum\:_{i}{w}_{ij}{x}_{i}+\:{b}_{j},\:\:\:\:\:\:\:{O}_{j}=f\left({net}_{j}\right)$$

In the Eq. (1), each neuron forms a weighted sum of its initial inputs plus a bias and passes it through a nonlinear activation *f*(.), for example sigmoid, tanh, ReLU. This is how features such as lags, seasonality flags, etc. are transformed layer by layer into a demand prediction^20^.

Error signal (“delta”) for output and hidden neurons (backward-propagation), for illustration, the common sigmoid-activation case can be written as follows:

Output layer:

2 $$\:{\delta\:}_{j}={O}_{j}\left(1-{O}_{j}\right)\left({t}_{j}-{O}_{j}\right)$$

Hidden layer:

3 $$\:{\delta\:}_{j}={O}_{j}\left(1-{O}_{j}\right){\sum\:}_{k}{\delta\:}_{k}{w}_{jk}$$

In the above, t_j_, is the target and O_j_ is the neuron’s output, $\:\delta\:$ then measures how much a neuron contributed to the overall error, thereafter, propagating it backward from the output to the hidden layers.

#### Support Vector Machines (SVMs)

Support Vector Machines are commonly used for classification, while Support Vector Regression extends the same margin-based principle to regression problems. The data points that determine this hyperplane are referred to as the support vectors. SVMs learn a function that’s as “flat” as possible while allowing small errors within an ε–tube. The inputs are then mapped to a high-dimensional feature space via a kernel, therefore a linear model in that space corresponds to a nonlinear model in the original space. In the context of urban water demand studies, SVR/SVMs are a common baseline and has often been chosen explicitly for near real-time demand forecasting, they are often improved further by hybrids e.g., SVR + Adaptive Fourier Series^24^.

SVM model in feature space (linear here, nonlinear there)

4 $$\:f\left(x\right)=\left[w,\:\varnothing\:\left(x\right)\right]+b$$

In the equation above, $\:\varnothing\:\left(.\right)$ maps the input to a feature space and w, b is then learnt. As a starting point, the model learns a flat function in a feature space that would for example predict demand from the predictors i.e. lags, seasonality, etc.

ε–insensitive SVR with slack variables (primal form)

5 $$\:{w,b\:\{{\xi\:}_{i}^{+},{\xi\:}_{i}^{-}\:\}}^{min}\:\:\:\:\:\frac{1}{2}{\left|\left|w\right|\right|}^{2}+C\frac{1}{n}\sum\:_{i=1}^{n}\left({\xi\:}_{i}^{+}+{\xi\:}_{i}^{-}\right)$$

Subject to:

$$\:{y}_{i}-\left[w,\:\varnothing\:\left({x}_{i}\right)\right]-b\le\:\epsilon\:+{\xi\:}_{i}^{+}$$

$$\:\left[w,\:\varnothing\:\left({x}_{i}\right)\right]+b-\:{y}_{i}\le\:\epsilon\:+{\xi\:}_{i}^{-}$$

$${{\xi\:}_{i}^{+},\xi\:}_{i}^{-}\ge\:0,\:\:\:\:\:i=1,\dots\:\dots\:.,n$$

This optimization therefore enforces flatness $\:\left({\left|\left|w\right|\right|}^{2}\right)$ while allowing deviations beyond the $\:\epsilon\:$-tube through the slack variables $\:{\xi\:}_{i}^{+}$ and $\:{\xi\:}_{i}^{-}$, balanced by the penalty parameter C. In that set-up, intuitively $\:\epsilon\:$ sets a no-penalty band whereas C controls the tolerance to violations.

Dual/kernelized predictor and common kernel

6 $$\:f\left(x\right)={\sum\:}_{i=1}^{n}\left({\alpha\:}_{i}-\:{\alpha\:}_{i}^{*}\right)K\left({x}_{i},\:x\right)+b,\:\:\:\:\:\:\:\:\:{K}_{RBF}\left({x}_{i},{x}_{j}\right)=\mathrm{exp}\left({-\gamma\:\left|\left|{x}_{i}-{x}_{j}\right|\right|}^{2}\right)$$

In the above equation, only support vectors have non-zero coefficients $\:\left({\alpha\:}_{i}-\:{\alpha\:}_{i}^{*}\right)$. Using RBF kernel introduces smooth nonlinear responses controlled $\:\gamma\:$^42,43^.

#### Linear Regression (LR)

Linear regression is a model that fits a weighted sum of predictors like for example: lagged demand, seasonal dummies, in order to explain the target. It’s often thought of as transparent, fast, and a standard baseline for demand/quality studies^44,45^. The model is fundamentally guided by the equation:

Model:

7 $$\:{\hat {\mathrm{y}}}=XZ+\epsilon$$

Least-squares estimate:

8 $${\hat {\mathrm{Z}}}=\mathrm{arg}{min}_{Z}{\left|\left|y-XZ\right|\right|}_{2}^{2}={\left({X}^{T}X\right)}^{-1}{X}^{T}y$$

The model can be presented in such a way that it captures residues or noise, which in that case would be plus ε.

#### Ensembles of Trees (EoT)

Ensembles of trees combines many trees in order to improve accuracy and stability the output. Bagging/Random Forests reduce variance by averaging decorrelated trees, whereas Boosting adds trees sequentially in order to correct residuals. Both families are widely applied in WRM and water-quality prediction^46^.

Bagging predictor:

9 $$\:{\hat {\mathrm{y}}}\left(x\right)=\frac{1}{B}\sum\:_{b=1}^{B}{T}_{b}\left(x\right)$$

With trees T_b_ grown on bootstrap samples.

Squared-error boosting (stagewise):

10 $$\:{F}_{m}\left(x\right)={F}_{m-1}\left(x\right)+v{h}_{m}\left(x\right),\:\:\:\:\:\:\:\:\:\:{r}_{i}^{\left(m\right)}={y}_{i}-\:{F}_{m-1}\left({x}_{i}\right)$$

Whereby each small tree h_m_ fits residuals r^(m)^ and 0 < $\:v$ ≤ 1is the learning rate.

#### Principal Component Analysis (PCA)

Principal Component Analysis is an unsupervised tool that rotates predictors into orthogonal components ordered by variance. It is often used before model training to reduce collinearity and dimension e.g., we capped PCA at 95% variance retained in the model training^47,48^.

Given centred X,

11 $$\:Covariance\:S=\frac{1}{n-1}{X}^{T}X$$

12 $$\:then\:the\:Eigen-decomposion:S=V\varLambda\:\:{V}^{T},\:\:PC\:scores\:are\:Z=XV$$

In the equation above, columns of V are principal directions, Z are the component scores used as inputs to a downstream regressor after having retained enough components to explain the desired variance.

### Data sources and processing

#### Rain data

**Fig. 3:**
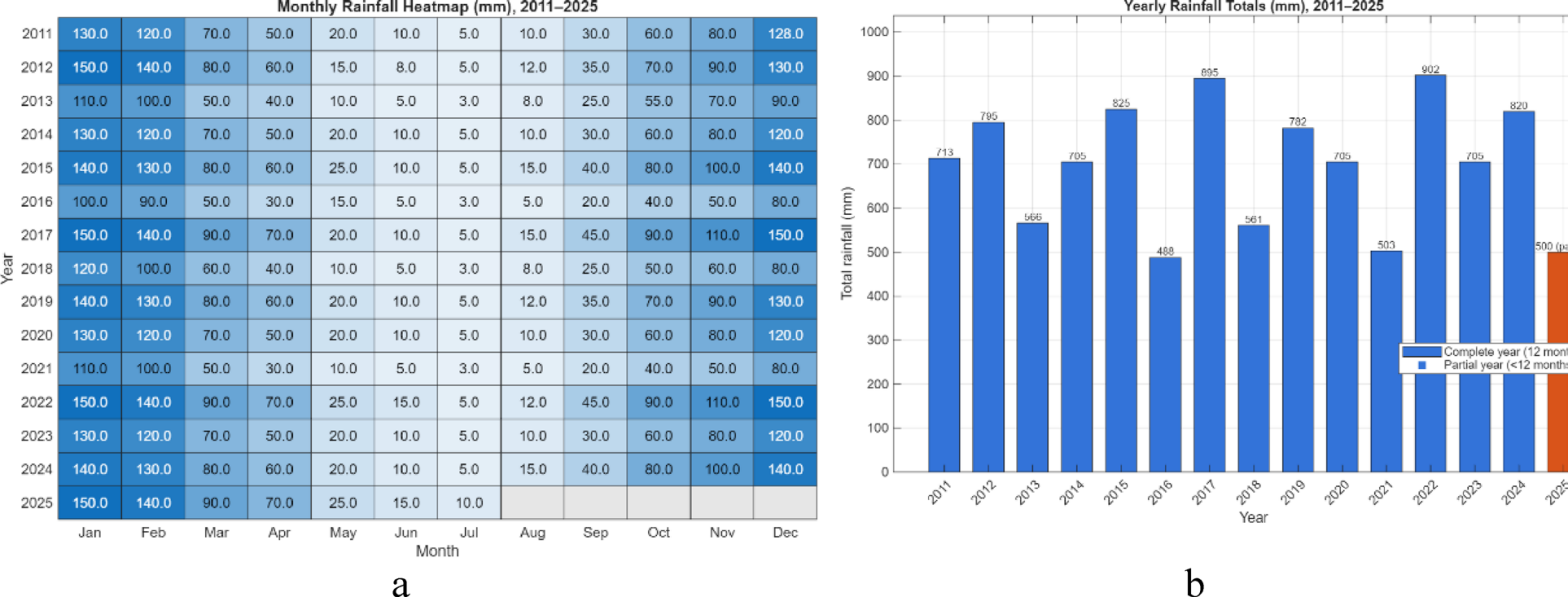
(**a**) Rainfall data heatmap, (**b**) trends analysis.

The rainfall shown in Fig. 3 was compiled, at a monthly resolution for 2011–2025 and thereafter treated as a univariate time series. In this context, “leak-safe” means that feature engineering, PCA, and scaling are recomputed inside each training fold, and all predictors stop strictly at month t–1 for a forecast at month t, preventing any information from the future leaking into model training. The dates were then parsed to datetime, sorted, and checked for gaps or duplicates. In order to capture temporal structure without leakage, we built past-only features: lags 1–12, trailing 3/6/12-month means, and month-of-year seasonality encoded with sine/cosine pairs. Rows whose predictors were incomplete (early months before enough history accumulated) were dropped. The train window was set to 2011–2022 and the test window between 2023 and 2025 and thereafter, evaluated models against persistence and seasonal-naïve baselines before producing 36-month forecasts by recursive rollout (feeding predictions back into lagged features). This follows established practice for seasonal energy/water-like loads where same-cycle effects and careful, past-only engineering are critical^49^.

#### Water consumption data

**Fig. 4:**
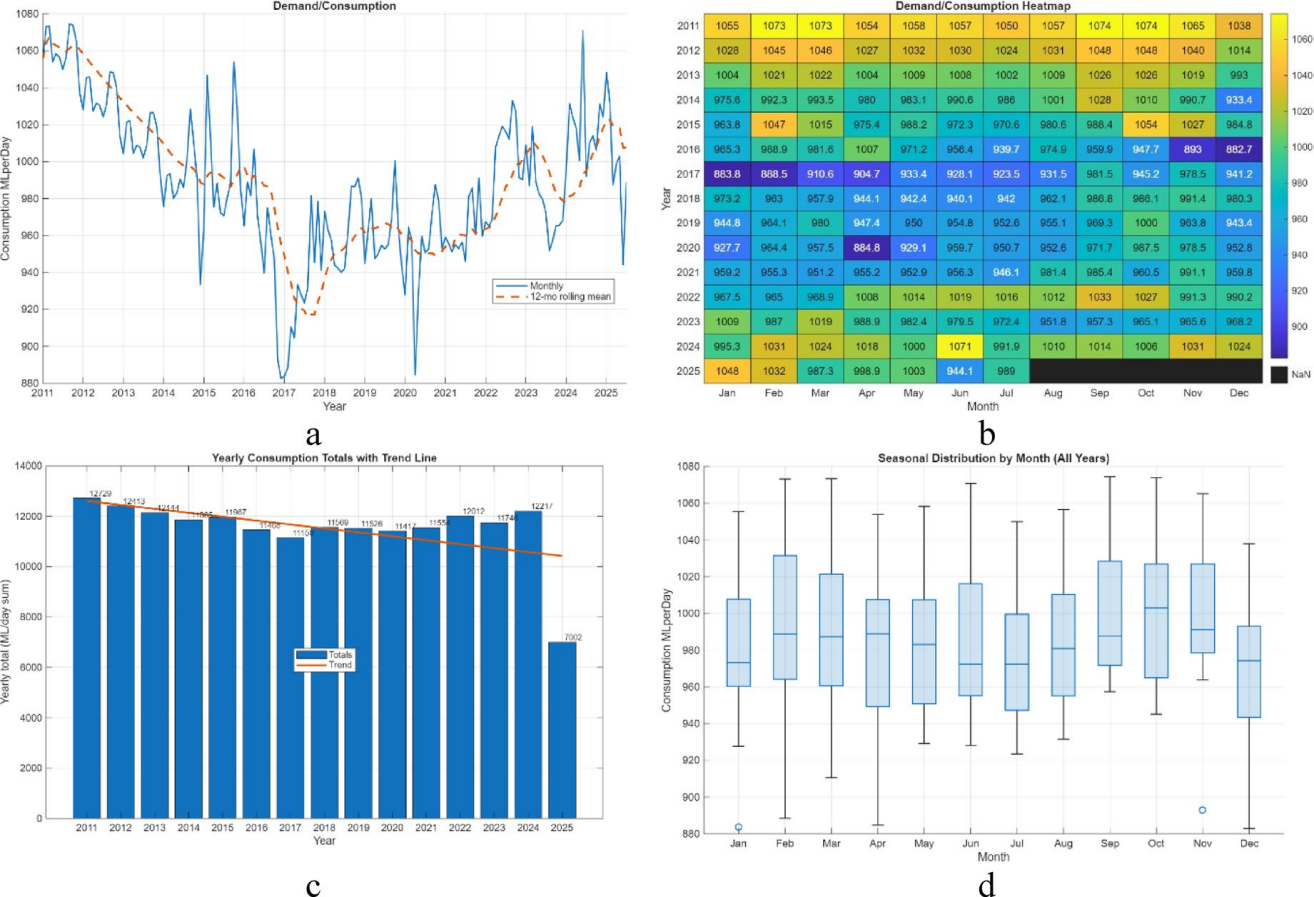
(**a**) Monthly consumption with trailing means, (**b**) Consumption/Demand heatmap, (**c**) Yearly trends analysis, (**d**) Seasonal distribution.

Monthly potable-water consumption for the City of Ekurhuleni (2011–2025) was also compiled at a monthly resolution as shown in Fig. 4. We applied the same leakage-safe pipeline: parse and sort dates, remove exact duplicates, inspect obvious errors, built lags 1–12 and trailing 3/6/12-month statistics, add month sine/cosine and a simple calendar trend; and retain only rows with complete past-only history. Training and validation were performed using MATLAB (5-fold CV with PCA equal to 95% variance when used) at a ratio of 80/20 (80% training and 20% testing). We reported RMSE, MAE, MAPE, R^2^, and normalized RMSE, and compared skill against persistence, seasonal-naïve, and climatology baselines. This mirrors guidance from comparative studies that show tree ensembles and kernel methods often outperform plain linear models for residential demand once lagged demand and simple calendar effects are included^50^.

#### Demographic information

Census-style attributes were assembled from the EA-type and water-access tables shown in the study. For each census year, we computed the share of households by EA category and the fraction with tap-water access. The generated trends were then used in-line with the above prediction to discuss prospects. The approach aligns with the literature’s guidance to tailor inputs to the forecasting horizon: socio-economic and service-access factors are more informative for medium-term (monthly, multi-year) planning than they are for very short-term control^51^.

The general ML process choices in terms of cross-validation, feature standardization inside training folds, and clear held-out testing, follow standard ML pipeline practice^52^.

### Feature engineering and variable selection

#### Rainfall

Data handling: we read the monthly series, then parsed date to datetime, sorted and removed duplicates. The rows were kept only when all past-only predictors were available which automatically trims the first 11–12 months.

Features:

- Autoregression lags, t-1,…,t-12.
- Trailing statistics (past-only windows), 3/6/12 month moving mean and sum with windows ending at t-1.
- Seasonality, month-of-year encoded as $\:\mathrm{s}\mathrm{i}\mathrm{n}(2\pi\:m/12)$ and $\:\mathrm{c}\mathrm{o}\mathrm{s}(2\pi\:m/12)$ in order to capture annual cyclicity without inflating dimensionality.
- Calendar trend, integer year for mild long-run drift.

Splits and baselines: we trained the model using the 2011–2022 data, using fivefold cross-validation (with PCA set at 95%) and tested using the 2023–2025 data, then produced 36-month forecasts by recursive rollout (feeding predictions back to construct future lags). Baselines were persistence (next month = last month), seasonal-naïve (this month = same month last year), and monthly climatology (historical mean by month). This mirrors monthly hydromet practice where seasonality and lag memory dominate and where leakage control is essential^53^.

#### Demand/consumption

Data handling: we ingested the city’s monthly potable consumption series, standardized column names, parsed/sorted dates, and then removed exact duplicates. Outliers were inspected but not winsorized unless clearly erroneous so as to preserve operational realism.

Features:

- Autoregression lags, t-1,…,t-12.
- Trailing statistics (past-only), moving mean/sum over 3/6/12 months (windows end at t-1).
- Seasonality, sin/cos month terms.
- Calendar trend, year index.
- Optional exogenous (low-frequency) inputs: EA-type shares and tap-water access were aligned on the monthly grid via last-observation-carried-forward, so each month inherits the most recent census snapshot. We retained these only if they improved cross-validated error.

*Variable selection*.

The selection was kept pragmatic and reproducible:

- Rule-based prefilter (before modelling).
  Enforced no leakage, only past-dependent features.
  Dropped rows lacking full lag history.
  Preferred sin/cos for seasonality over 11 one-hot month dummies (compact, smooth, less collinearity).
  Avoided redundant moving windows.
- Model-based diagnostics (during/after CV).
  Used tree/ensemble importance to identify consistently weak predictors and then prune those that do not reduce CV-RMSE.
  In cases whereby the PCA was used, it was applied inside folds (so selection respects CV and avoids leakage).
  Re-checked test-set baselines after any selection change to ensure claimed gains are real.
- Parsimony for forecasting.
  We retained the smallest feature set that (a) passes baselines on the test window and (b) remains stable across sensitivity splits. This is consistent with monthly-planning case studies that balance accuracy with maintainability in operations^54^.

This was the engineering/selection workflow used to deliver the data-leak-safe features, rigorous baselines, and 36-month forecasts reported in the results. To support replication in other municipalities, the MATLAB scripts used for data-leak-safe feature engineering, cross-validation and model training are available from the corresponding author upon reasonable request.

### Model training strategy

As previously stated, the model training strategy followed a data-leak-safe, time-aware pipeline: ingesting and cleaning the monthly tables, engineering past-only predictors, splitting the data by time, and training with cross-validation. This approach mirrors standard ML practice for supervised regression pipelines^55,56^.

After defining the data partitions and validation procedure, several regression families were compared, including linear and lasso models, SVM with Gaussian kernel, tree-based learners, Gaussian Process Regression and compact neural networks, rather than relying on a single hypothesis class. We required every candidate to beat three simple but informative baselines on the test set: persistence (y_t_ ≈ y_t−1_), seasonal-naïve ((y_t_ ≈ y_t−12_) and monthly climatology (historical mean by month). The chosen model maximized test-set skill vs. these baselines and minimized RMSE/MAE while maintaining reasonable MAPE and R^2^. Using multiple naive comparators is standard in short-term utility-load work because it guards against picking a model that looks good only relative to another ML model.

In order to ensure robustness, a simple linear recalibration was done where necessary then re-evaluated on the sealed test set. For forward planning we produced 36-month forecasts by recursive rollout: predict *t* + 1, appended it to the series to build future lags, then predict *t* + 2, and so on. This matches how short-term energy and utility forecasts are commonly produced when models rely on lagged targets.

### Evaluation metrics and validation

Below are various metrics and validation techniques in evaluating the models’ performance^57–62^.

- Error-based metrics.

Mean Absolute Error.

13 $$\:\left(MAE\right)=\frac{1}{n}\sum\:_{i=1}^{n}|{y}_{i}-{{\hat {\mathrm{y}}}}_{i}|,\,\, \text{lower is better}.$$

Mean Squared Error

14 $$\:\left(MSE\right)=\frac{1}{n}\sum\:_{i=1}^{n}{\left({y}_{i}-{{\hat {\mathrm{y}}}}_{i}\right)}^{2}$$

Root Mean Squared Error.

15 $$\:\left(RMSE\right)=\sqrt{MSE},\;\;\text{RMSE penalizes larger errors more strongly}$$

Mean Absolute Percentage Error

16 $$\:\left(MAPE\right)=\frac{100\%}{n}\sum\:_{i=1}^{n}\left|\frac{{y}_{i}-{{\hat {\mathrm{y}}}}_{i}}{{y}_{i}}\right|$$.

Normalized RMSE and CVRMSE

17 $$\:\left(NRMSE\right)=\frac{RMSE}{\stackrel{-}{y}}, \quad CVRMSE=100\% \times \frac{RMSE}{\stackrel{-}{y}}$$

b. Correlation/efficiency metrics.

Pearson correlation (r) and Coefficient of determination (R^2^)

18 $$r = \frac{{\sum\nolimits_i {({y_i} - \bar y)({{\hat y}_i} - \bar{\hat{y}})} }}{{\sqrt {\sum\nolimits_i {{{({y_i} - \bar y)}^2}} } \sum\nolimits_i {{{({{\hat y}_i} - \bar{\hat{y}})}^2}} }}$$

19 $$\:{R}^{2}=1-\frac{{\sum\:}_{i}{\left({y}_{i}-\:{{\hat {\mathrm{y}}}}_{i}\right)}^{2}}{\sum\:_{i}{\left({y}_{i}-\stackrel{-}{y}\right)}^{2}}$$.

c. Baselines and skill scores.

To ensure that the models outperformed simple references, three baselines were evaluated:

- Persistence (predict the last value). A standard benchmark: the Persistence Index (PI) form compares model SSE to persistence SSE:

20 $$\:PI=1-\frac{\sum\:{\left({y}_{i}-{{\hat {\mathrm{y}}}}_{i}\right)}^{2}}{\sum\:{\left({y}_{i}-{y}_{i-1}\right)}^{2}}$$

Positive values indicated improvement over persistence. The “skill” was summarized as percentage improvement in RMSE relative to a baseline *B*.

21 $$\:{Skill}_{\%}\left(B\right)=100\: \times \:\left(1-\frac{{RMSE}_{model}}{{RMSE}_{B}}\right)$$

A positive skill means the model beats the baseline whereas a negative skill means the baseline is better.

## Results and discussion

### Rainfall prediction

As shown in Table 2, numerous models were trained, across all the results the bagged-tree ensemble (model 10) was the clear winner on the 2023–2025 test window, posting RMSE 9.13 mm, MAE 6.85 mm, R^2^ 0.96, MAPE 16.3%. It cut test RMSE by ~ 27% relative to the next best model, the Fine Tree (12.54 mm; R^2^ 0.93) and by ~ 34% relative to Linear Regression (13.82 mm, R^2^ 0.92).

Boosted Trees performed similarly to the Fine tree (12.75 mm, R^2^ 0.93), while the linear family (Robust/Plain/Stepwise/Interactions) clustered between 13 and 16 mm RMSE with R^2^ ≈ 0.89–0.92, indicating that much of the seasonal/lag structure is linearly explainable but variance reduction from ensembles still paid off. The two neural networks were competitive (13.43–14.22 mm, R^2^ ≈ 0.91–0.92) but did not surpass the best tree models.

SVM (Linear) and SVM (Fine Gaussian) lagged (20.23–20.72 mm, R^2^ 0.82–0.83), and the SVM Kernel variant was the weakest by far (41.03 mm, R^2^ 0.29, MAPE 128%). MAPE rose sharply for several models because percentage errors inflate in very dry months, the RMSE/MAE and R^2^ tell the more reliable outlook. Overall, the ensemble models, especially bagging, generalized best and captured peak-trough seasonality with the smallest errors; therefore, the bagged-tree ensemble was selected for forecasting.

As an additional point, across the 5-fold cross-validation, the bagged-trees rainfall model showed stable performance, with validation RMSE of 17.44 and validation R^2^ 0.86. The competing tree and linear models showed overlapping but consistently weaker scores as initially highlighted. This modest spread indicates that the ranking in Table 2 is not driven by a single favourable split and that the bagged-trees model provides robust out-of-sample skill for monthly planning that is usable to practitioners.

**Table 2:** Rainfall models’ result.

Model type	Validation				Testing				
	RMSE	MSE	*R* ^2^	MAE	MAE	MAPE %	MSE	RMSE	*R* ^2^
Fine Tree	17.98	323.2	0.85	12.81	8.86	18.19	157.2	12.54	0.93
Linear Regression	19.53	381.29	0.82	15.48	11.76	44.43	190.92	13.82	0.92
Interactions Linear Regression	19.22	369.47	0.83	15.53	12.77	37.62	267.59	16.36	0.89
Robust Linear Regression	20.83	434.04	0.8	15.06	10.69	31.33	179.31	13.39	0.92
Stepwise Linear Regression	19.4	376.19	0.83	15.61	12.76	37.56	267.73	16.36	0.89
Linear SVM	25.71	661.11	0.69	18.69	14.72	35.12	409.28	20.23	0.83
SVM (Fine Gaussian)	13.13	172.53	0.92	9.85	15.25	72.17	429.32	20.72	0.82
SVM Kernel	39.17	1534.21	0.29	31.87	34.17	128.08	1683.29	41.03	0.29
Ensemble (Boosted Trees)	15.26	232.75	0.89	11.24	9.44	22.24	162.58	12.75	0.93
Ensemble (Bagged Trees)	17.44	304.02	0.86	12.66	6.85	16.29	83.36	9.13	0.96
Narrow Neural Network	15.56	242.03	0.89	11.89	10.22	25.48	180.4	13.43	0.92
Bilayered Neural Network	14.29	204.19	0.91	9.75	9.52	25.94	202.24	14.22	0.91

**Fig. 5:**
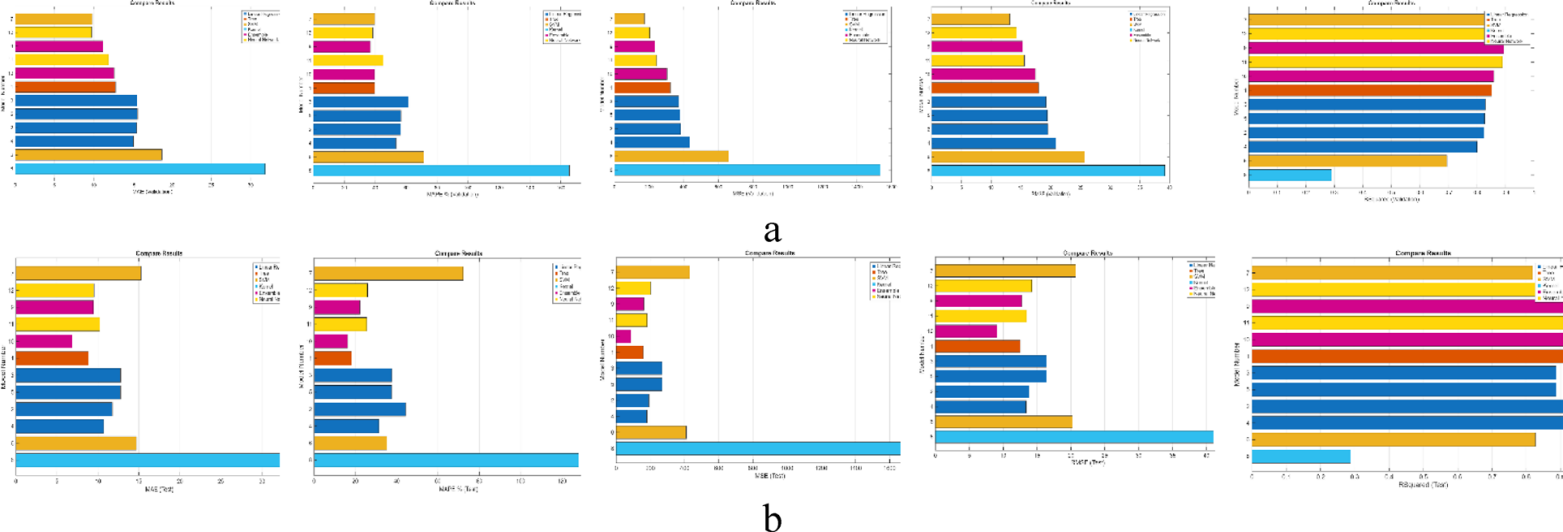
(**a**) validation results (MAE, MAPE, MSE, RMSE, R^2^), (**b**) Testing results (MAE, MAPE, MSE, RMSE, R^2^).

Figure 5 shows a graphical representation of the results in Table 2.

**Fig. 6:**
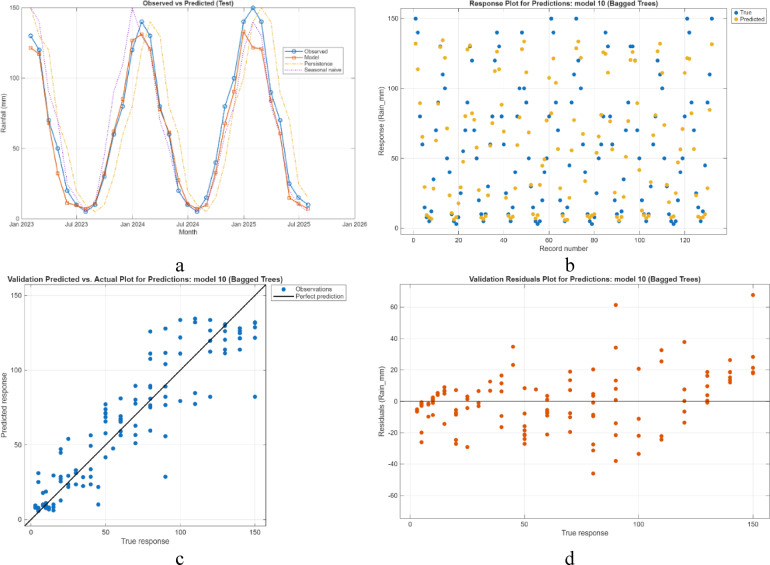
(**a**) Observed Vs Predicted (test), (**b**) Response plot, (**c**) Validation predicted vs. actual plot, (**d**) Validation Residuals plot.

Figure 6a. (Observed vs. Predicted with baselines) the chosen model (orange line) tracks the observed peaks and troughs across 2023–2025 much more closely than persistence and seasonal-naive. It catches the summer peaks (Dec–Feb) and the winter minima (Jun–Aug) with smaller phase error. Occasional under-prediction appears at the very top of the wet season, but the amplitude and timing are aligned overall, which matches with the high R^2^. Validation Predicted vs. Actual (scatter, with 1:1 line) in Fig. 6c, points lie close to the 1:1 line.

At very high totals (> ~ 120–140 mm) you can see a slight shrinkage toward the mean (predictions a bit below the line). That is typical of bagging as it reduces variance and can slightly dampen the extremes. It helps stability and is often a good trade-off for monthly planning. Validation Residuals vs. True response Residuals in Fig. 6d, centre near zero across most of the range, with spread increasing as totals rise. The mild funnel shape signals heteroskedasticity, errors are naturally larger in wet months. That aligns with hydromet behaviour and is acceptable as long as bias stays small. Response Plot in Fig. 6b, for Predictions Predicted values span the observed range and don’t collapse toward the mean, which confirms the model is using seasonal and lagged structure rather than learning a flat climatology.

**Fig. 7:**
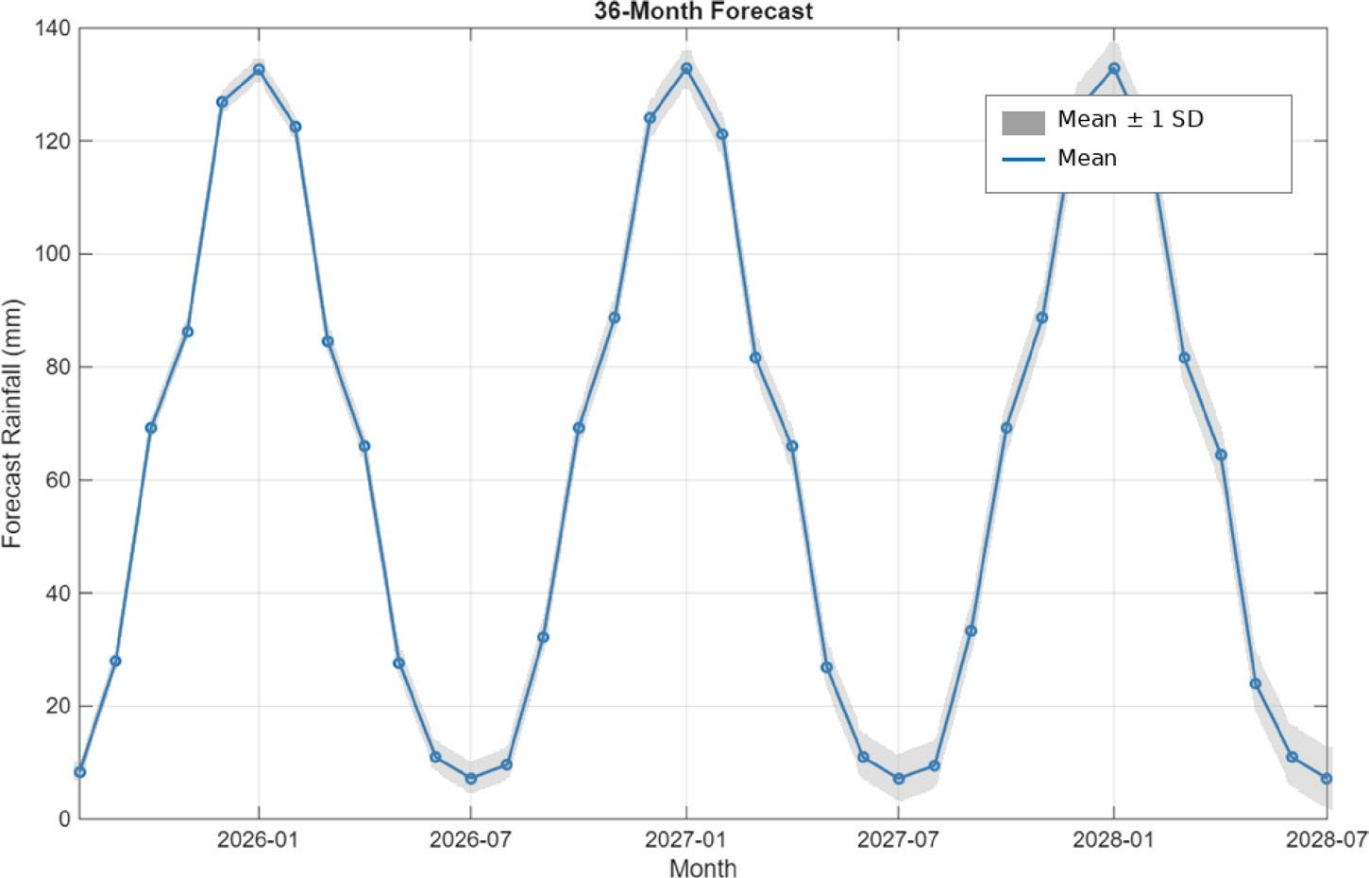
36-month rainfall forecast (the solid line shows the 36-month mean rainfall forecast; the shaded ribbon shows Mean ± 1 standard deviation, giving an approximate uncertainty band around the seasonal cycle).

Figure 7 shows the 36-month forecast/prediction, as seen the wet-season peaks cluster in Dec–Feb with totals around 124–133 mm (Dec ≈ 124–127, Jan ≈ 132–133, Feb ≈ 118–122). Dry-season minima occur in Jun–Aug (≈ 7–11 mm), with a small recovery by September and a clear ramp into October–November (≈ 69–89 mm) ahead of the summer peak. These trends are consistent with the previous patterns and broadly in terms of the rainfall patterns in South Africa.

The uncertainty ribbon remains relatively narrow and nearly constant across the 36-month horizon, indicating that most of the forecast variance comes from seasonal swings rather than explosive growth in long-range uncertainty.

### Water demand/consumption

Table 3 shows the results from the models based on the water demand/consumption within the municipality. On the test set results, the best performers were the Gaussian Process models: Matern 5/2 and Matern 3/2 which achieved R^2^ ≈ 0.756 and 0.751 with RMSE ≈ 17.1 and 17.2 ML/day and MAPE ≈ 1.39–1.41%. Bagged Trees followed (R^2^ ≈ 0.679, RMSE ≈ 19.6, MAPE ≈ 1.56%). Linear SVM and plain Linear Regression were close behind (R^2^ ≈ 0.655 and 0.637; RMSE ≈ 20.3 and 20.8; MAPE ≈ 1.71% and 1.61%). The custom tree landed mid-pack (R^2^ ≈ 0.505, RMSE ≈ 24.3). The pattern showed water demand in Ekurhuleni has smooth nonlinear structure that GPR captures well, while a strong linear component kept the linear baselines competitive. Importantly, for the top models the test meets or exceeds validation R^2^, which suggests stable generalization given the small sample size.

At the other end, Fine Tree and Stepwise Linear lagged (R^2^ ≈ 0.229 and 0.236; RMSE ≈ 30; MAPE ≈ 2.43–2.50%). Kernelized SVM variants underperformed, the SVM Kernel scored R^2^ ≈ 0.085 (RMSE ≈ 33.0; MAPE ≈ 2.55%) and the Fine Gaussian SVM slipped slightly negative on R^2^, which hinted at poor kernel scaling or overfit for the data volume. Boosted Trees was the clear outlier with negative R^2^ (≈ − 0.66), high RMSE (≈ 44.5), and the largest MAPE (≈ 3.98%), which points to a mismatch in depth/learning rate or simply too few observations for boosting to shine. Model 3, the Matérn-5/2 GPR model, was therefore selected because it achieved the lowest test RMSE, MAE and MAPE among the demand models.

**Table 3:** Consumption/Demand results.

Model type	Validation					Testing				
	RMSE	MSE	*R* ^2^	MAE	MAPE %	RMSE	MSE	*R* ^2^	MAE	MAPE %
Fine Tree	30.47	928.20	0.34	22.68	2.32	30.33	920.13	0.23	23.53	2.43
Matern 3/2 GPR	24.85	617.47	0.56	17.57	1.80	17.25	297.40	0.75	13.74	1.41
Matern 5/2 GPR	25.05	627.66	0.55	17.70	1.81	17.05	290.76	0.76	13.50	1.39
Linear Regression	23.17	536.70	0.62	16.37	1.66	20.82	433.54	0.64	15.63	1.61
Stepwise Linear	36.08	1301.78	0.07	24.49	2.49	30.18	911.04	0.24	24.38	2.50
Linear SVM	26.68	711.98	0.49	20.28	2.07	20.28	411.43	0.66	16.47	1.71
Fine Gaussian SVM	36.50	1332.39	0.05	29.29	3.00	34.82	1212.38	−0.02	26.07	2.72
Boosted Trees	48.73	2374.44	−0.69	43.49	4.38	44.50	1980.45	−0.66	39.18	3.98
Bagged Trees	25.14	632.04	0.55	18.55	1.90	19.58	383.27	0.68	15.06	1.56
SVM Kernel	34.59	1196.65	0.15	27.40	2.80	33.05	1092.02	0.08	24.45	2.55
Custom Tree	26.48	701.18	0.50	20.12	2.06	24.30	590.56	0.50	20.48	2.13

**Fig. 8:**
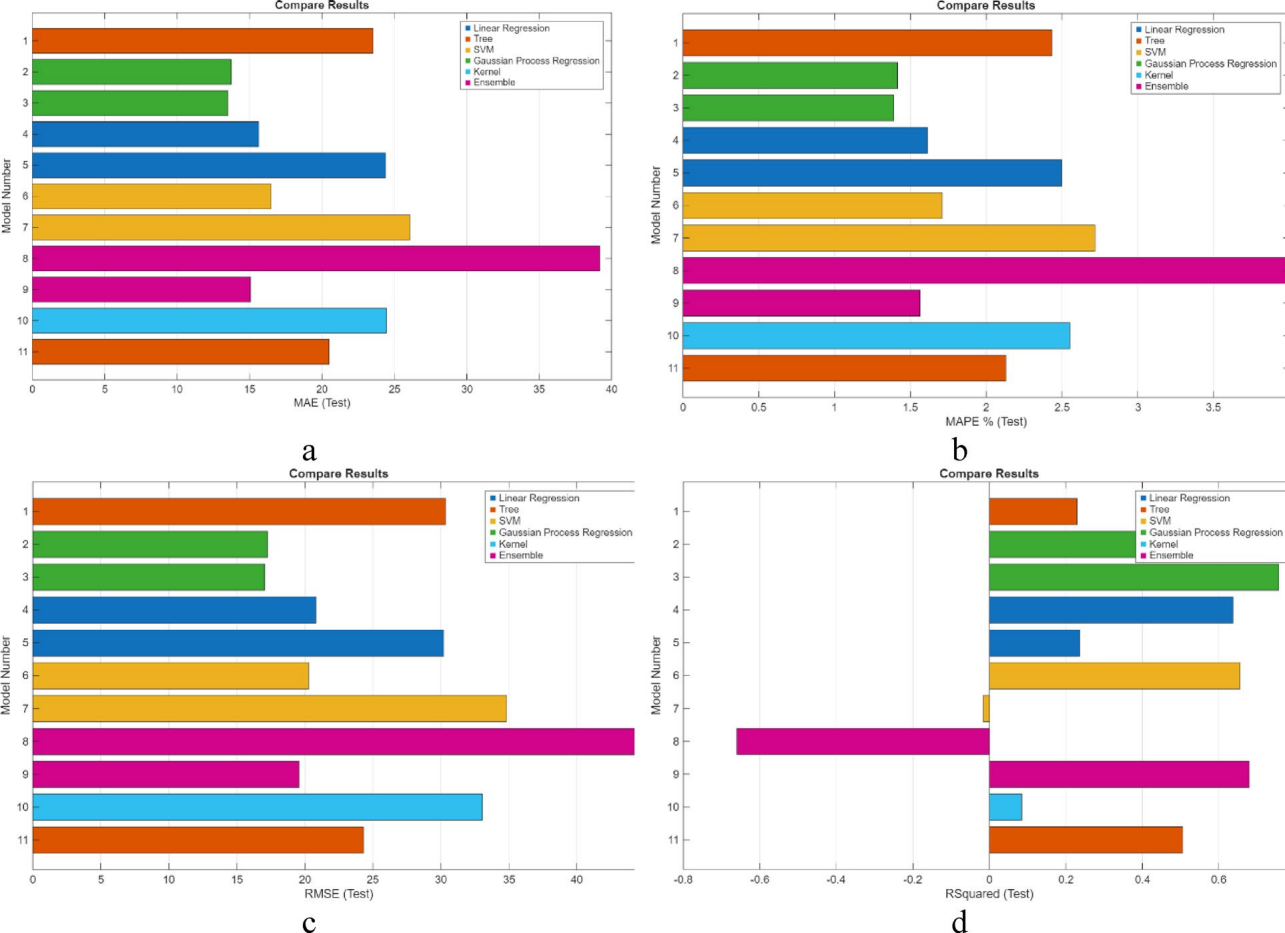
(**a**) Test MAE, (**b**) Test MAPE, (**c**) Test RMSE, (**d**) R^2^.

Figure 8 shows a graphical representation of the test results of the models.

**Fig. 9:**
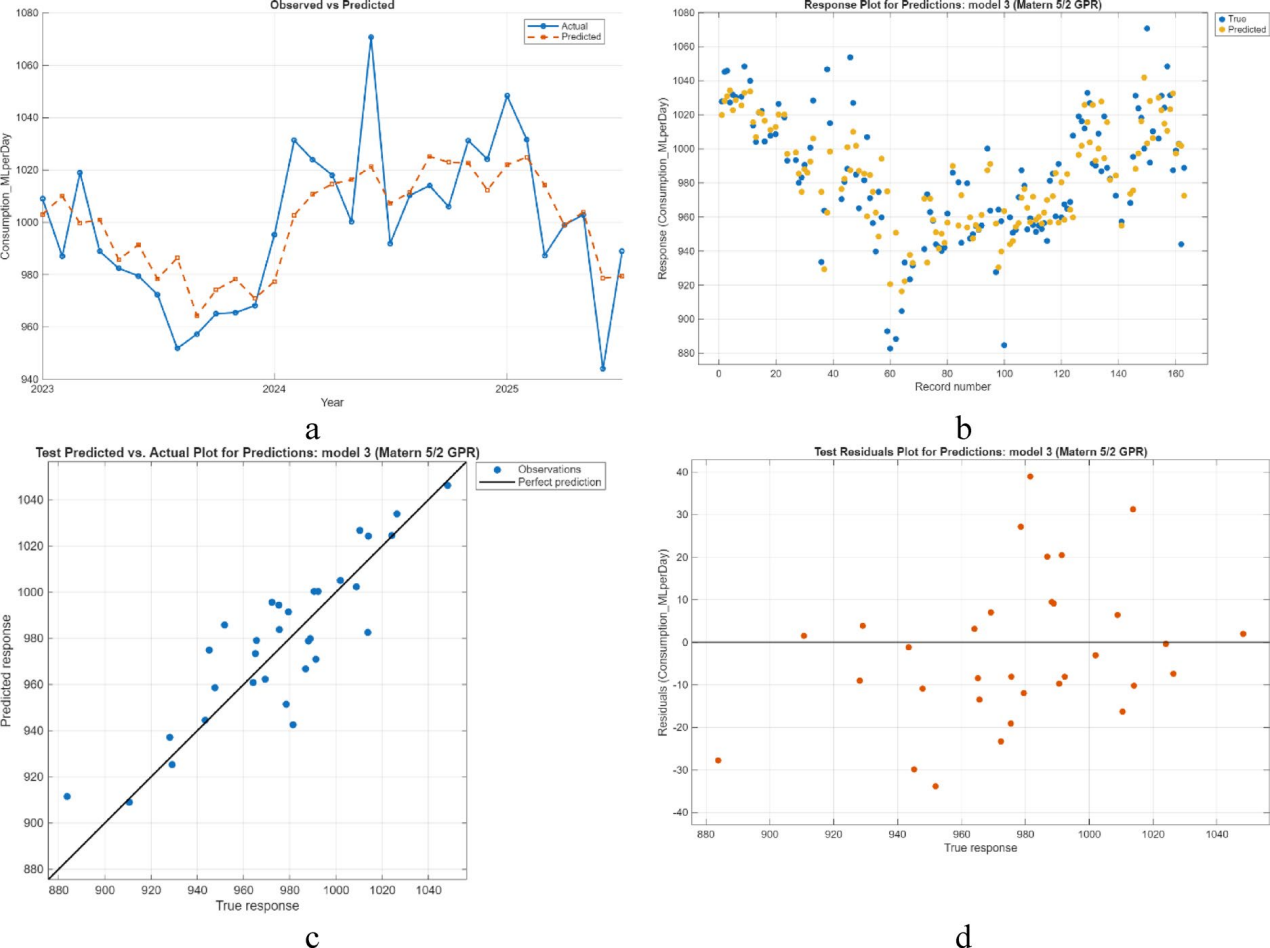
(**a**) Observed vs. predicted test plot, (**b**) response plot, (**c**) test predicted vs. actual plot, and (**d**) test residuals plot for demand/consumption.

In Fig. 9a. (Observed vs. Predicted (test, 2023–2025) provides an overview of the model, the dashed prediction tracks the timing of rises and falls well and preserves month-to-month continuity. The amplitudes are damped: sharp spikes (e.g., the 2025 peak) are under-predicted and sharp troughs are nudged up.

That shrinkage is typical of GPR with smooth kernels and partly explains the moderate, yet positive test R^2^. Test Predicted vs. Actual (1:1 scatter) shown in Fig. 9c, shows the points are aligned along the 1:1 line with a slope slightly below one, indicating a small under-prediction bias at the top end and a slight over-prediction at the low end.

Figure 9d (Test residuals vs. true response), shows the residuals are centred around zero with heteroskedasticity: dispersion widens between ~ 980–1020 ML/day (busy months).

There is no strong curvature pattern, which supports the kernel’s smoothness choice; however, the negative cluster around higher volumes confirms the mild high-end bias noted above. Figure 9b shows the response plot (all records), the predictions follow the low-frequency envelope of the series rather than collapsing to a flat mean, which is consistent with the kernel’s ability to learn smooth nonlinear trends from lagged features and seasonality.

Model 3 is a pragmatic choice for monthly planning as it captures the seasonal trajectory and month-to-month dynamics (good MAE/NRMSE), with an interpretable, correctable amplitude bias.

The 36-month projection shows a stable band of demand/consumption roughly ~ 995–1 025 ML/day, with gentle intra-year undulations and no strong long-run drift, meaning the model expects stable base demand with only small month-to-month ripples and no clear upward or downward trend. There’s a brief crest in the first third of the horizon, a soft dip through mid-2027, then a modest recovery, all of these movements stay within the same tight range, so the year-to-year differences are minor.

**Fig. 10:**
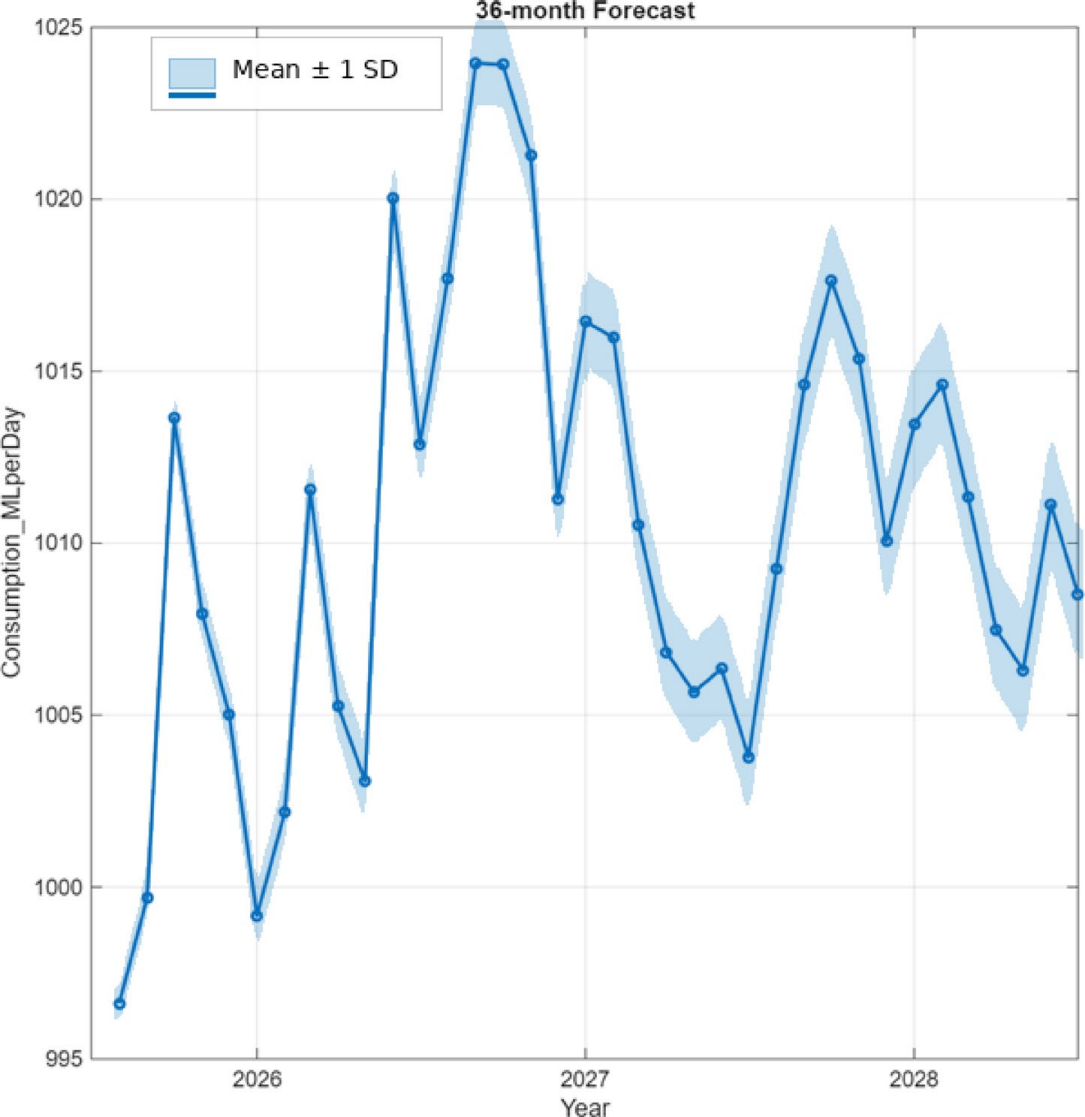
36-month demand/consumption forecast (The solid line shows the 36-month mean demand forecast; the shaded ribbon shows Mean ± 1 standard deviation of forecast uncertainty (“Mean ± 1 SD”).

The Mean ± 1 SD band thickens slightly further into the horizon as multi-step uncertainty accumulates, but remains within a tight range, reinforcing the interpretation of a stable base-load band rather than a drifting trend (Fig. 10).

The same smoothness that makes the Matérn-5/2 GPR attractive for monthly planning also means that very sharp spikes and dips in consumption are partially damped. From an infrastructure perspective this is conservative for average-month decisions but can be risky if used as a single input for sizing or stress-testing storage, pumping or transfer capacity under rare peaks or deep droughts. In practice, the stable 36-month outlook should be combined with explicit stress tests (e.g. applying safety factors or overlaying historical peak scenarios) when assessing whether existing infrastructure can tolerate short-term extremes.

### Enumeration area (EA) type and water access analysis

**Fig. 11:**
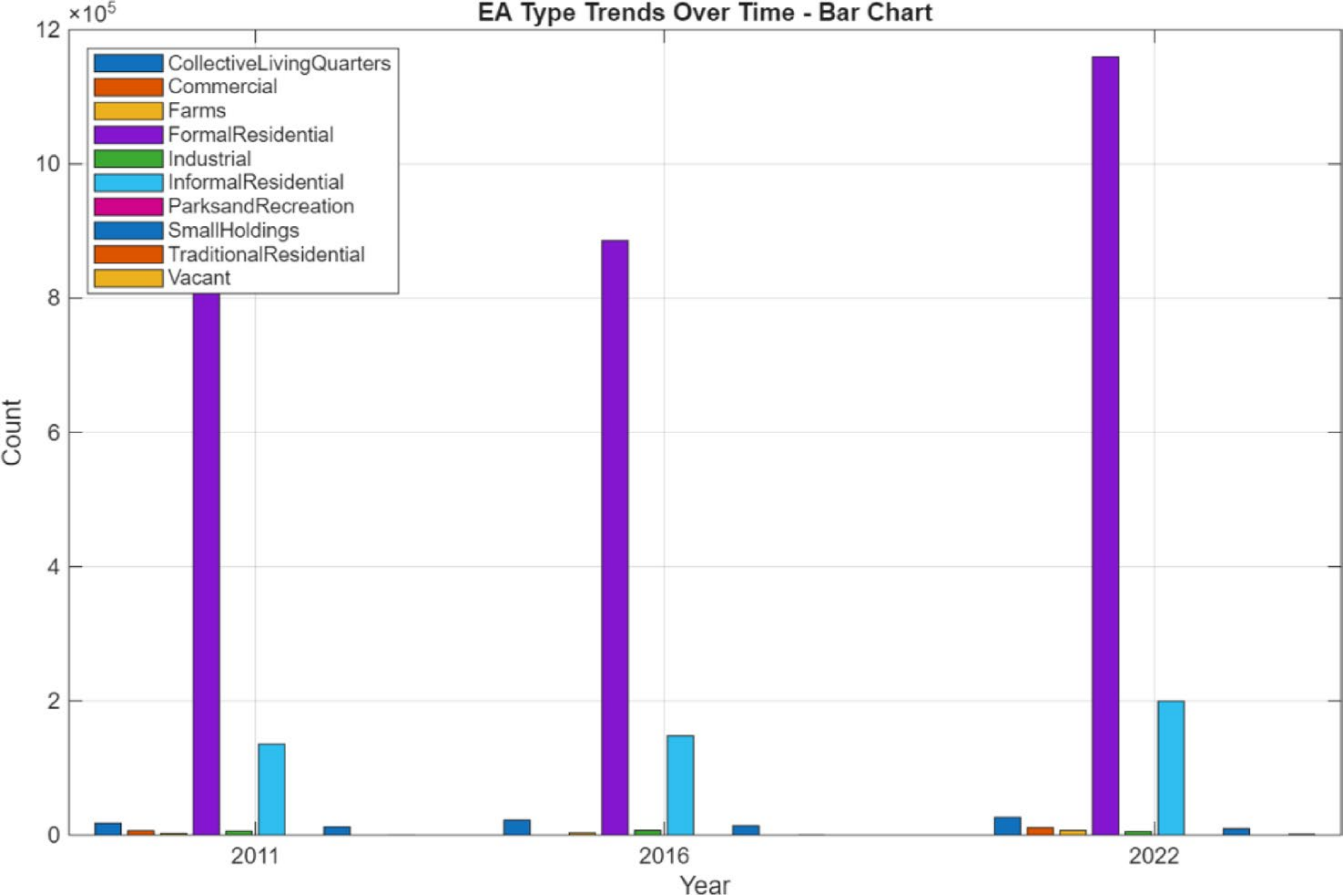
Ekurhuleni enumeration area type population growth over time.

Figure 11 shows a trend of population by enumeration area over time, specifically during the period of 2011 to 2022 based on the census data. As seen, the formal residential category dominates and has grown strongly from 2011 and especially by 2022 (as shown in Fig. 11). With this, informal residences have also increased across the three censuses (light-blue bars rising), while small categories like: commercial, industrial, farms/smallholdings, parks & recreation, traditional residential, collective living quarters, and vacant, remain comparatively minor and mostly stable. This trend indicates a metro that is densifying and formalising its housing stock: more formal dwellings by 2022, with informal areas still present and growing but at a much smaller scale than the formal base.

**Fig. 12:**
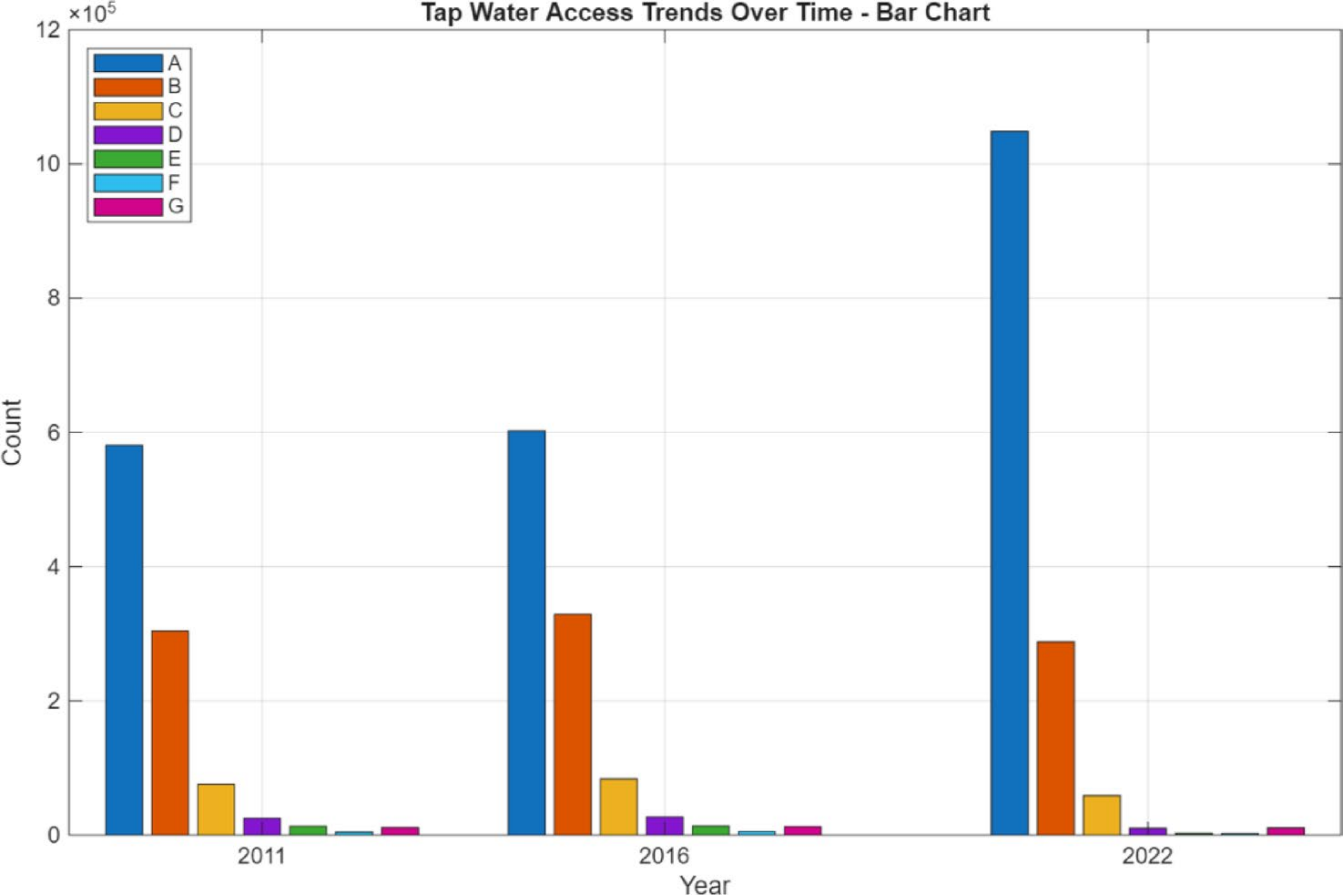
Ekurhuleni water access over time.

Figure 12 shows access to water over various categories: A – inside the dwelling, B – inside the yard, C – on community stand with a distance less than 200 m from the dwelling, D - to community stand with a distance less than 200 m and 500 m from the dwelling, E - to community stand with a distance less than 500 m to 1000 m from the dwelling, F - on community stand with a distance greater than 1000 m from the dwelling, G – no access. From the trend below, a larger formal residence growth generally shifts consumption toward a higher and more predictable base load (more metered, in-dwelling connections, indoor appliances), while growth in informal areas adds variability and localized peak stresses. This suggests that population growth and improved household water access are likely to increase the demand for reliable municipal water services over time.

Based on the demand forecast and supply-demand history, Rand Water appears able to meet the projected municipal requirement over the forecast horizon; however, continued municipal infrastructure investment may still be required to maintain reliability under seasonal rainfall variability and changing demand patterns.

In this initial implementation, the EA-type and water-access categories are used descriptively to interpret the stability of the predicted base load rather than as direct predictors in the demand model. A natural next step is to incorporate slow-moving demographic indicators (for example, the share of formal residences or the share of households with in-dwelling or yard taps) as exogenous features and quantify their effect sizes on consumption. That would allow the demographic trends in Figs. 11 and 12 to be tested directly within the forecasting framework rather than only being used qualitatively.

### Implications for infrastructure and plicy

#### Infrastructure and service equity

The forecasts point to a stable municipal demand band with gentle month-to-month undulations (~ 995–1 025 ML/day) and clear seasonal rainfall cycles (summer peaks, winter minima). For rainfall, the bagged-trees model slightly underestimates the highest wet-season totals because of the variance-reduction effect of bagging and the monthly aggregation. This bias is acceptable for bulk-supply and reservoir-operations planning at monthly scale, but it means the forecasts should not be used in isolation for detailed flood-risk or stormwater design. In those applications, managers should combine the monthly projections with separate extreme-value or event-based analyses that explicitly target peak intensities.

The demographic trends show continued growth of formal residential areas alongside smaller but persistent growth in informal areas, with steady gains in A/B tap-water access categories. Together, these patterns frame a planning problem that is less about explosive growth and more about reliability, equity and targeted upgrades over the next 36 months, for example prioritising pressure management and leak repair in high-consumption township belts such as Tembisa and Kathorus and focusing reservoir and trunk-main upgrades along supply corridors like the Mapleton-Benoni system.

Simple demographic-demand scenario analysis can then be built by combining the forecast demand band with projected EA shares and tap-access levels when assessing where upgrades and pressure management should be prioritised.

#### Forecasting practice and operations

Implemented together, these measures can lead to: pressure/NRW reduction, operational buffering, targeted service upgrades, and routine re-forecasting which in turn raises effective system yield and reliability without new capacity, advancing SDG 6 by increasing the share of households with safely managed drinking water and sanitation and, operationally by stabilizing and modestly improving litres-per-capita-per-day across currently under-served EA categories.

These results support a policy focus on reliability, non-revenue water reduction, targeted equity upgrades and disciplined, baseline-aware decision-making rather than wholesale capacity expansion. In practical terms this means using the monthly forecasts as one of the standard inputs into reservoir-operation rules, pressure-management and leakage-reduction programmes, treating sharp policy changes such as new restriction stages or tariff adjustments as explicit scenario shocks layered onto the baseline, and periodically retraining the models as new data come in, while framing decisions within explicit uncertainty bands. For instance, if the 36-month demand outlook shows several consecutive years with consumption near the upper forecast band during below-normal rainfall seasons, the city can pre-emptively negotiate higher Rand Water allocation bands, intensify active leakage control in the highest-loss zones, and bring forward reservoir or pumpstation upgrades in the Mapleton and Palmiet supply corridors. In essence, Ekurhuleni can use the 36-month demand envelope and uncertainty bands to set medium-term Rand Water purchase envelopes and reservoir target levels and to time leakage-reduction and pressure-management projects against the forecast seasonal peaks.

## Conclusion and recommendations

### Conclusion

This study developed a reproducible monthly forecasting framework for rainfall and municipal water demand in Ekurhuleni using past-only, data-leak-safe features and rigorous baseline comparisons. By combining simple but carefully designed lags, trailing statistics and seasonal terms with cross-validated machine-learning models, we showed that it is possible to obtain decision-grade forecasts. For rainfall, a bagged-trees ensemble captured the summer–winter cycle with low error on the sealed 2023–2025 test window. For demand, a Matérn-5/2 Gaussian Process reproduced the month-to-month dynamics with small absolute error and a stable 36-month outlook, while explicitly benchmarking performance against persistence, seasonal-naïve and climatology baselines. This gives Ekurhuleni’s planners a transparent tool that can be audited, updated as new data arrive and embedded into routine planning cycles.

In the South African context, where many municipalities still rely on ad-hoc spreadsheets or untested rules of thumb, the contribution is less about using “exotic” models and more about demonstrating a clear, leak-aware workflow that respects data limitations and focuses on reliability, NRW reduction and equity rather than premature capacity expansion. The results provide a starting point for integrating data-driven forecasts into Rand Water procurement planning, reservoir operation, pressure management and targeted service upgrades, while leaving room for future extensions to higher-frequency data and richer exogenous drivers.

There are limits and clear avenues to improve robustness. The consumption model slightly damps extremes, so adding calibrated uncertainty bands and testing a small set of slow exogenous drivers (restriction level, tariff shifts, holiday markers) would strengthen risk communication without overfitting. Finally, the demographic trend toward more formal residential areas and greater in-dwelling tap access suggests a stable but gradually more metered base load.

Because rainfall and water demand in Gauteng are shaped by climate variability and gradual socio-economic change, these models should not be treated as fixed. Instead, the framework is intended to be adaptive: parameters are re-estimated as new years of data accumulate, model skill is re-checked against simple baselines after major droughts, floods or policy shifts and forecast bands are updated to reflect any detected non-stationarity in the underlying processes.

### Recommendations for future work

- Probabilistic forecasts - extend point forecasts to quantiles (e.g., quantile regression forests or GPR predictive intervals) would support operational planning.
- Applying richer exogenous drivers - test low-frequency external predictors that are operationally available in Ekurhuleni, such as restriction levels, tariff changes, outage days, holiday flags, temperature degree-days, rainfall anomalies and large development approvals, and then add only those that improve sealed-test error to keep models lean.
- Hierarchical/segment models - where the data allows, build zone-level or customer-class models and reconcile to the system total. This in turn can expose local hotspots and improve intervention targeting.
- Robust validation - complementing the current CV with blocked time-series CV as well as rolling-origin back tests to stress test generalization under different structural regimes (e.g., pre-/post-intervention periods) can help in further improving the validation robustness.
- Demographic microanalysis - between 2011 and 2016, the Ekurhuleni Municipality had more wards (112) compared to the initial 101 in the year 2011. Meaning between these years and thereafter, some wards split while others merged, as such conducting a demographic micro analysis at ward level was difficult due to the border changes, with more data, future work would allow for better analysis.

## Data Availability

the data is available upon reasonable request, and the data is also publicly available on the relevant department’s websites.
